# 

**Published:** 1885-12

**Authors:** 


					﻿Entered at the Post Office at Austin. Toras, as Second Class Man Matter.
Vol. 1.]
DECEMBER, 1885.
[No. 6.
DANIEL'S
MEDICAL
JOURNAL
F. E. DANIEL, M. D., Editor and Publisher, AUSTIN. TEXAS.
MOBTHERK OFFICE 1217 FILBERT CTREE? : :iILA7X:.! HIA.
EIGEXE VOX HOECKMAXV, PHIXTEK, AVSTJX, TEXAS
				

